# Prognosis and subtype analysis of left ventricular noncompaction in adults: A retrospective multicenter study

**DOI:** 10.1002/clc.23991

**Published:** 2023-02-13

**Authors:** Yunfei Feng, Lili Ning, Jing Zhang, Huaigen Wang, Hanzhao Zhang, Ruochen Zhang, Zhengrong Deng, Yajuan Ni, Yulan Ye, Aiqun Ma, Yun Zhang, Tingzhong Wang

**Affiliations:** ^1^ Department of Cardiovascular Medicine Xi'an Jiaotong University Medical College First Affiliated Hospital Xi'an Shaanxi Province China; ^2^ Department of Radiology Xi'an Jiaotong University Medical College First Affiliated Hospital Xi'an Shaanxi Province China; ^3^ Department of Cardiovascular Medicine Shaanxi Provincial People's Hospital Xi'an Shaanxi Province China; ^4^ Department of Cardiovascular Medicine Xi'an Jiaotong University Second Affiliated Hospital Xi'an Shaanxi Province China; ^5^ Department of Cardiovascular Medicine Xi'an Gaoxin Hospital Xi'an Shaanxi Province China

**Keywords:** ejection fraction, left ventricular noncompaction, prognosis, subtype

## Abstract

**Background:**

Left ventricular noncompaction (LVNC) is a heterogeneous myocardial disorder with an uncertain prognosis. There was a lack of studies on LVNC subtypes at present. This study sought to identify the prognosis of the overall population of LVNC and to describe the distribution of different subtypes and compare their prognosis.

**Hypothesis:**

Patients with different subtypes of LVNC may have different prognoses.

**Methods:**

Patients who fulfilled the Jenni criteria and/or Petersen criteria were included. Major adverse cardiovascular events (MACE) were defined as a combination of heart failure (HF) hospitalization and all‐cause mortality.

**Results:**

A total of 200 patients from four hospitals were included. The mean age at diagnosis was 48.2 years, and 61.5% of the patients were male. Left ventricular ejection fraction (LVEF) < 50% was present in 54% of the patients. Over a mean retrospective time period of 22.2 months, 47 (23.5%) patients experienced MACE. Age (hazard ratio [HR] 1.03; 95% confidence interval [CI] 1.01–1.06; *p* = .004), LVEF < 50% (HR 2.32; 95% CI 1.09–4.91; *p* = .028) and ventricular tachycardia/ventricular fibrillation (HR 2.17; 95% CI 1.08–4.37; *p* = .03) were significantly associated with the risk of MACE. The most common subtype was dilated LVNC (51.3%), followed by benign LVNC (21.3%) and LVNC with arrhythmias (10.5%). Patients with dilated LVNC had significantly increased cumulative incidence of MACE, HF hospitalization, and all‐cause mortality (*p* < .05).

**Conclusions:**

Age, LVEF < 50%, and ventricular tachycardia/ventricular fibrillation were independent risk factors for prognosis of LVNC. The most common subtype was dilated LVNC, which had a worse prognosis.

## Graphical abstract

Prognosis and subtype analysis of left ventricular ejection fraction.

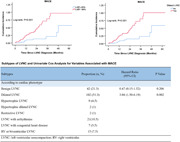

## 1 INTRODUCTION

Left ventricular noncompaction (LVNC) is a heterogeneous myocardial disorder characterized by two distinct layers: a thin compacted outer layer and a thick noncompacted (NC) inner layer, with excessive trabeculations and deep intertrabecular recesses.^1^ LVNC has no specific clinical presentation. It can occur at any age, and symptoms can range from asymptomatic to heart failure (HF), lethal arrhythmias, sudden cardiac death (SCD), stroke, or other thromboembolic events.^2^, ^3^ LVNC was first described by Grant in 1926 and divided into eight subtypes by Towbin et al. in 2015, including benign, dilated, hypertrophic, dilated hypertrophic, restrictive, right/biventricular, LVNC with arrhythmias, and LVNC with congenital heart disease (the specific definition of each subtype is in the Supporting Information: Table S1).^1^ An increasing number of studies focused on the characteristics and prognosis of the overall population of LVNC, but there was a lack of research on different subtypes. Thus, we conducted a multicenter study in four cardiac centers from Northwest China (1) to investigate the clinical characteristics and prognostic variables of LVNC; (2) to describe the distribution of different subtypes and compare their different prognosis.

## 2 METHODS

### 2.1 Study design and population

This was a multicenter, observational, retrospective, longitudinal cohort study. In four local hospitals in Xi'an, Shaanxi Province of Northwest China, we searched for patients with a possible or definite diagnosis of LVNC from January 1, 2010, to December 31, 2021. A total of 238 patients were recruited (144 at The First Affiliated Hospital of Xi'an Jiaotong University, 14 at The Second Affiliated Hospital of Xi'an Jiaotong University, 70 at Shaanxi Provincial People's Hospital, and 10 at Xi'an Gaoxin Hospital). The study was in accordance with the Declaration of Helsinki and approved by the hospital ethics committee. As part of the study, we contacted each patient to inform him/her of the retrospective chart review and obtain longitudinal information on survival and major adverse cardiovascular events (MACE). Considering the minimal risk, the requirement for informed consent was waived for the study.

The inclusion criteria were as follows: (1) age ≥ 18 years; (2) diagnosis of LVNC based on Jenni criteria and/or Petersen criteria.^4^, ^5^ The exclusion criteria were (1) incomplete clinical data (2) inability to contact the patient. The most common diagnostic method is based on the ratio of the thickness of the NC layer to that of the compacted (C) layer. The Jenni criteria were defined as NC/C ≥ 2 at the end of systole on echocardiography, and the Petersen criteria were defined as NC/C ≥ 2.3 at the end of diastole on cardiovascular magnetic resonance (CMR).

### 2.2 Data collection

All data were collected from the medical records. The clinical characteristics included demographics, comorbidities, symptoms at presentation, and treatment at baseline. The myocardial imaging data were collected through echocardiography and CMR. Left ventricular (LV) dysfunction was defined as LV ejection fraction (LVEF) < 50% on echocardiography because all the patients in the cohort underwent echocardiography. LV dilation was defined as^6^ LV end‐diastolic diameter >55 mm for males and >50 mm for females, and left atria (LA) dilation was defined as LA diameter >40 mm. For ECG data, arrhythmias included ectopic rhythms and conduction abnormalities. The corrected QT interval (QTc) was calculated by the Bazett's formula: QTc = QT/(RR^0.5^). The criteria for QTc prolongation were ≥460 ms for males and ≥450 ms for females.^7^ The voltage criteria for LV hypertrophy were calculated by the Sokolow index.^8^

### 2.3 Onset of symptoms and time of diagnosis

We carefully reviewed all information of the patients. We found that most patients had one or more cardiac‐related visits or routine physical examinations before the diagnosis of LVNC was confirmed. Often, echocardiography did not detect LVNC at the first encounter or first few encounters. Therefore, we defined “onset” as the time when the patient first had symptoms and visited the cardiology department. We defined “the time of diagnosis” as the time when the patient had a confirmed diagnosis of LVNC.

### 2.4 Outcomes

Information on the occurrence of clinical events was collected from the medical records, outpatient visits, and telephone enquiries. Retrospective study time is the time from the original diagnosis. Telephone contacts were conducted and completed in April 2022. The clinical endpoints of the study were (1) HF hospitalization: a composite of required hospitalization for new‐onset HF, acute decompensation of chronic HF; and (2) all‐cause mortality (cardiovascular mortality was included). MACE were defined as a combination of the two endpoints. We used MACE for our hazard models because of the low incidence of death. We did not observe the incidence of ventricular tachycardia (VT)/ventricular fibrillation (VF) or systemic embolism (stroke or peripheral thromboembolism).

### 2.5 Statistical analysis

Continuous variables were expressed as the mean ± SD and were compared among the groups using independent samples *t* tests. Categorical variables are expressed as the number of cases and proportions and were compared using the *χ* ^2^ test or Fisher's exact test, as appropriate. Analysis of the survival data was performed using the Kaplan–Meier method, and differences between the groups were assessed by the log‐rank test. Risk factors for MACE were analyzed by univariate and multivariate Cox regression analyses. The variables that were significantly associated with MACE on univariate analysis and those with considerable clinical impact were included in the multivariate model. The results were expressed as hazard ratio (HR) and 95% confidence interval (CI). A *p* value <.05 was considered statistically significant.

## 3 RESULTS

**Figure 1:**
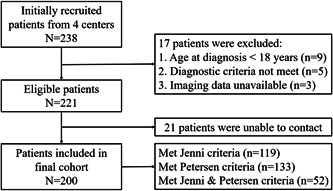
Flow chart of study population screening. CMR, cardiovascular magnetic resonance.

Initially, a total of 238 patients were recruited. 38 patients were excluded, 9 were younger than 18 years at the time of initial diagnosis, 5 did not meet the diagnostic criteria, 3 did not have imaging data, and 21 were unable to contact. Finally, 200 patients were enrolled in our study cohort (Figure 1). A total of 119 patients (59.5%) met the Jenni criteria and 133 patients (66.5%) met the Petersen criteria.

### 3.1 Baseline characteristics

#### 3.1.1 Clinical characteristics

The mean age at diagnosis was 48.2 years, and 61.5% of the patients were male. The mean time from the onset of symptoms to diagnosis was 2.52 years. The most common comorbidity was hypertension (29.5%), followed by dyslipidemia (20%), coronary heart disease (17%), and diabetes mellitus (12.5%). Only 4% of the patients had a definite family history of cardiomyopathy or SCD. The most common symptom was dyspnea (66%), and 10.5% of the patients were asymptomatic. For treatment, beta‐blockers were used in 77% of the patients, angiotensin receptor‐neprilysin inhibitor (ARNI) in 42.5%, angiotensin‐converting enzyme (ACE) inhibitors or angiotensin receptor blockers (ARB) in 39%, mineralocorticoid receptor antagonist (MRA) in 56% and sodium‐glucose cotransporter 2 (SGLT2) inhibitors in 6.5%. Additionally, 20.5% of the patients were on anticoagulation treatments at baseline. In addition to medication, 7% of the patients received treatment of cardiovascular implantable devices at or before diagnosis (Supporting Information: Table S2).

#### 3.1.2 Myocardial imaging data

Prior echocardiography was available in all patients. The mean LVEF was 48.17%, and LV dysfunction (EF < 50%) was present in 108 (54%) patients. LV dilation and LA dilation were present in 129 (64.5%) and 58 (29.2%) patients, respectively. Forty‐two (21%) patients had varying degrees of pulmonary hypertension and nine (4.5%) had LV thrombosis. CMR had been performed in 133 (66.5%) patients. The mean end‐diastolic noncompacta thickness was 14.35 mm, the mean end‐diastolic compacta thickness was 4.03 mm and the mean end‐diastolic NC/C ratio was 3.19 (Supporting Information: Table S2).

#### 3.1.3 ECG data

A total of 51.5% of the patients had at least one type of arrhythmia, including ventricular arrhythmias (37.5%), atrial flutter/atrial fibrillation (11%), left bundle branch block (10.5%) and atrioventricular block (7.5%). The ECG also showed LV hypertrophy by the voltage criteria in 14.5% of the patients and QTc prolongation in 29.5%. Repolarization abnormalities were present in as many as 65% of the patients (Supporting Information: Table S2).

### 3.2 Outcomes

Over a mean retrospective time period of 22.2 months, 47 (23.5%) patients experienced MACE (Supporting Information: Table S2). HF hospitalization occurred in 33 (16.5%) patients. There were 16 (8%) patients died, including 14 (7%) patients who died due to cardiovascular causes.

**Figure 2:**
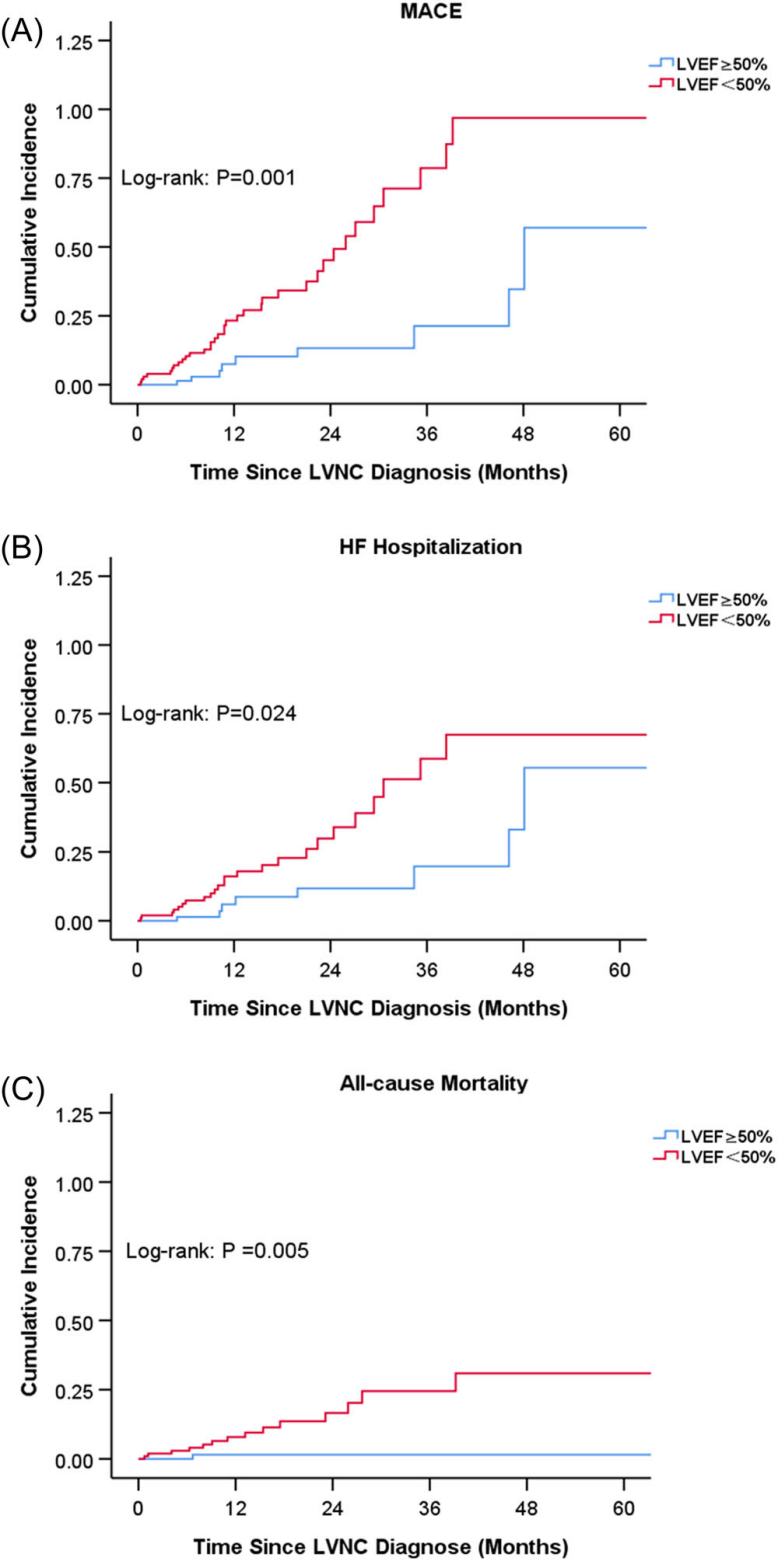
Kaplan–Meier curves for the cumulative incidence of (A) major adverse cardiovascular events (MACE), (B) heart failure (HF) hospitalization, and (C) all‐cause mortality stratified by left ventricular ejection fraction (LVEF).

Based on the number of MACE, we included four variables in the multivariable model (Table 1): including the variables that were significantly associated with MACE on univariate analysis (Supporting Information: Table S3) and those with considerable clinical impact. Age (HR 1.03; 95% CI 1.01–1.06; *p* = .004), LVEF < 50% (HR 2.32; 95% CI 1.09–4.91; *p* = .028) and VT/VF (HR 2.17; 95% CI 1.08–4.37; *p* = .03) were still significantly associated with the risk of MACE. Kaplan–Meier survival analysis showed EF < 50% was significantly associated with higher cumulative incidence of MACE, HF hospitalization, and all‐cause mortality (*p* < .05) (Figure 2).

**Table 1:** Multivariable Cox analysis for the variables associated with MACE.

Variables	Hazard ratio (95% CI)	*p* Value
Age	1.03 (1.01–1.06)	.004
LVEF < 50%	2.32 (1.09–4.91)	.028
Atrial flutter/atrial fibrillation	1.61 (0.56–4.63)	.052
Ventricular tachycardia/ventricular fibrillation	2.17 (1.08–4.37)	.03

Abbreviations: CI, confidence interval; LVEF, left ventricular ejection fraction; MACE, major adverse cardiovascular events.

### 3.3 LVNC subtypes

**Figure 3:**
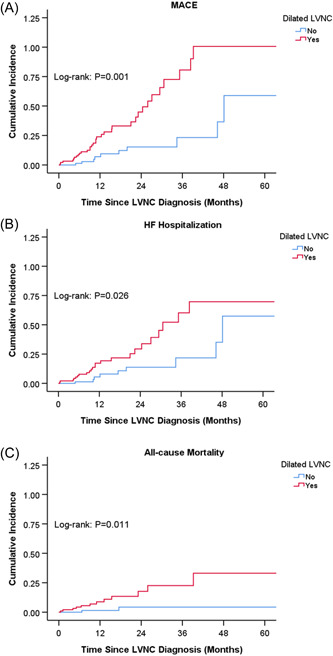
Kaplan–Meier curves for the cumulative incidence of (A) major adverse cardiovascular events (MACE), (B) heart failure (HF) hospitalization, and (C) all‐cause mortality stratified by dilated left ventricular noncompaction (LVNC).

We counted the proportion of different subtypes and evaluated their effects on MACE (Table 2). The most common subtype was dilated LVNC (51.3%), followed by benign LVNC (21.3%) and LVNC with arrhythmias (10.5%). Hypertrophic dilated LVNC and restrictive LVNC were rare. Noncompaction mainly affected LV (92.5%), including isolated apical noncompaction (40%) and mid‐basal noncompaction extent (52.5%). Dilated LVNC was significantly associated with a higher risk of MACE (HR 3.04; 95% CI 1.50–6.19; *p* = .002). Despite not achieving statistical significance, benign LVNC appeared to have a lower risk of MACE (HR 0.47; 95% CI 0.15–1.52; *p* = .206). By contrast, mid‐basal noncompaction extent was not associated with MACE, but significantly related to LV systolic function (Supporting Information: Table S4). Kaplan–Meier survival analysis showed that patients with dilated LVNC had significantly increased cumulative incidence of MACE, HF hospitalization, or all‐cause mortality (*p* < .05) (Figure 3).

**Table 2:** Subtypes of LVNC and univariate Cox analysis for variables associated with MACE.

Subtypes	*n* (%)	Hazard ratio (95% CI)	*p* Value
According to cardiac phenotype			
Benign LVNC	42 (21.3)	0.47 (0.15–1.52)	.206
Dilated LVNC	102 (51.3)	3.04 (1.50–6.19)	.002
Hypertrophic LVNC	9 (4.5)		
Hypertrophic dilated LVNC	2 (1)		
Restrictive LVNC	2 (1)		
with arrhythmias	21 (10.5)		
with congenital heart disease	7 (3.5)		
RV or biventricular LVNC	15 (7.5)		
According to noncompaction parts			
LV noncompaction	185 (92.5)		
Isolated apical	80 (40)	0.83 (0.45–1.52)	.547
Mid‐basal extent	105 (52.5)	1.10 (0.61–1.97)	.758
RV noncompaction	6 (3)		
Biventricular noncompaction	9 (4.5)	2.62 (0.94–7.36)	.06

Abbreviations: CI, confidence interval; LVNC, left ventricular noncompaction; MACE, major adverse cardiovascular events; RV, right ventricular.

## 4 DISCUSSION

In this multicenter study, we identified the following findings: (1) for adult LVNC, age, LV dysfunction (LVEF < 50%), and VT/VF were independent risk factors for the incidence of MACE; (2) the most common subtype in our patients was dilated LVNC, which had a worse prognosis.

### 4.1 Baseline characteristics of LVNC

In our cohort, the mean age at diagnosis was 48.2 years, the majority of patients were male, the most common comorbidity was hypertension, and more than half of the patients had cardiac structural and functional abnormalities. These baseline characteristics were comparable to those of other single‐center, multicenter, or different regional studies.^9^, ^10^, ^11^, ^12^, ^13^, ^14^ A total of 10.5% of the patients were asymptomatic at presentation, and they were usually identified after referral because of the presence of cardiac abnormalities on routine physical examination. Notably, the mean time from the onset of symptom to the correct diagnosis was 2.52 years. During this period, some patients might undergo unnecessary repetitive or excessive examinations. In addition to the lack of awareness of LVNC, it seems that the most commonly used echocardiography is not optimal for the detection of LVNC. In our cohort, of the 133 patients diagnosed by CMR, only 52 patients (39.1%) were detected by echocardiography. A previous study also showed that echocardiography only detected LVNC morphology in a small minority of patients (27%) who fulfilled the CMR criteria.^15^

### 4.2 Risk factors for prognosis

In previous large cohort studies and meta‐analyses, decreased LVEF was considered to be significantly associated with increased mortality and adverse clinical outcomes.^12^, ^13^, ^16^, ^17^ Our finding was concordant with the existing evidence. EF < 50% was an independent risk factor for MACE. Compared to patients with LVEF ≥ 50%, patients with EF < 50% were associated with a 3.19‐fold higher risk of MACE. Additionally, the presence of VT/VF at baseline was another independent risk factor for MACE in our study, which was similar to previous reports.^18^, ^19^, ^20^ On univariate analysis, structural abnormalities (e.g., LV dilation, LA dilation) and LV thrombosis appeared to predict a worse prognosis. This showed that the worse the patient's cardiac structure and function at the time of diagnosis, the worse the prognosis.

The degree of noncompaction, including the NC/C ratio, number of NC segments, mass, and thickness of noncompaction, was not considered to be associated with the prognosis of LVNC in most studies.^14^, ^20^, ^21^ We calculated the hazard ratios of the noncompaction degree for MACE in the patients who met the Peterson criteria (*n* = 133), and came to a consistent result: there was no significant correlation between the degree of noncompaction and the incidence of MACE. However, other previous studies showed that the NC/C ratio, segments, and mass of noncompaction were independent predictors of LV systolic dysfunction.^22^, ^23^ Therefore, the effect of the degree of noncompaction on prognosis needs further investigation.

### 4.3 Subtypes of LVNC

LVNC was previously divided into eight subtypes according to cardiac phenotype.^1^ However, some of these types were only discussed based on case reports or pediatric patients, so we investigated the subtypes of LVNC in adults. Our findings showed that dilated LVNC was the most common subtype. Compared to other subtypes, dilated LVNC was significantly associated with increased incidence of MACE, HF hospitalization, and all‐cause mortality. A few studies also compared the prognosis of dilated LVNC with that of dilated cardiomyopathy (DCM). The prognosis of dilated LVNC versus DCM in adults was controversial. Gerard et al. showed that dilated LVNC had a poorer prognosis^24^; however, Stanton et al. reported that the outcomes in LVNC were similar to those in DCM.^21^ This might be related to different inclusion criteria and study populations. In pediatric patients, there was a consensus that the long‐term outcome of dilated LVNC was worse than those of matched children with DCM.^25^, ^26^ A total of 21.3% of our patients were benign LVNC, which might indicate a better prognosis. On the basis of this subtype, some cardiologists have stated that LVNC was not a cardiomyopathy but a benign and normal variant.^27^ It also suggests that LVNC and LVNC cardiomyopathy should be two different terminology or different pathological stages of the same disease? At present, it is not clear whether LVNC is a separate cardiomyopathy or merely a congenital or acquired morphological trait shared by many phenotypically distinct cardiomyopathies.^28^, ^29^, ^30^ It was classified as a distinct genetic cardiomyopathy by the American Heart Association^31^; however, the European Society of Cardiology defined it as unclassified cardiomyopathy.^32^ Reasonable classification may help us to solve the above problems.

With regard to mid‐basal noncompaction extent, which has been previously associated with increased overall mortality compared with isolated apical noncompaction,^12^ it did not confer an increased risk of MACE but was an independent predictor of LV systolic dysfunction in our cohort. This finding could be explained by the short retrospective time and low incidence of MACE.

### 4.4 Study limitations

Several important limitations were present in our study. First, this was an observational study with limitations inherent to the retrospective design. The relatively short retrospective time led to a low incidence of events. We will continue to follow up with these patients to observe the occurrence of long‐term adverse events. There were 21 out of 221 patients who were unable to be contacted, their uncertain outcomes might affect the event rate. In the future, we will conduct prospective studies to minimize missing rate through systematic follow‐up. Second, although we have noticed that LVNC had different subtypes, we did not perform a comparison in depth between benign LVNC and matched healthy population, dilated LVNC and matched DCM, and so on, which should be focused on in future studies. It is very important to accurately distinguish between normal variation and pathological cardiomyopathy. Third, we lacked information on gene testing and did not analyze genotype‐phenotype correlations. Similar to the clinical phenotype, the genotype of LVNC is also heterogeneous and most cases of LVNC are associated with mutations in the same genes associated with other types of inherited cardiomyopathies, such as dilated cardiomyopathy and hypertrophic cardiomyopathy.^16^ It would be meaningful to see if the morphologic patterns have any correlation to genetic subsets, for example, genes that encode cytoskeleton, sarcomere, ion channels, etc. Fourth, pathologic findings were not available to better understand the specific patterns of the LVNC due to ethical concerns.

## 5 CONCLUSIONS

In adult patients with LVNC, age, LVEF < 50% and VT/VF were independent risk factors for prognosis. The most common subtype was dilated LVNC, which had a worse prognosis. As Jefferies, J. L. said, the absence of a cohesive and unified approach to the terminology, diagnosis, surveillance, and management of LVNC continues to be a source of frustration for providers around the global.^33^ Therefore, subtype analysis, more effective detection methods will help to solve the above problems.

## Supporting information

Supporting information.

## Data Availability

The data that support the findings of this study are available on request from the corresponding author. The data are not publicly available due to privacy or ethical restrictions.
