# Supplementary material for: Prognosis and subtype analysis of left ventricular noncompaction in adults: A retrospective multicenter study
Source: Clin Cardiol. 2023 Feb 13;46(4):390–6. doi: 10.1002/clc.23991 (PMC10106667; doi:10.1002/clc.23991)
Supplement: Supplementary file 1 — Supporting information. [file CLC-46-390-s001.docx]

**Supplementary Material**

1. **Specific definition of each subtype:**

**Benign LVNC**: normal left ventricular size and wall thickness with preserved systolic and diastolic function.

**Dilated LVNC**: concomitant left ventricular dilation and systolic dysfunction.

**Hypertrophic LVNC**: left ventricular thickening, usually with asymmetrical septal hypertrophy, in addition to diastolic dysfunction and hypercontractile systolic function.

**Hypertrophic dilated LVNC**: left ventricular thickening, dilation, and depressed systolic function at presentation.

**Restrictive LVNC:** left atrial or biatrial dilation and diastolic dysfunction.

**Right ventricular or biventricular LVNC**: hypertrabeculation of the right ventricle or both the right and left ventricles.

**LVNC with arrhythmias**: normal left ventricular size and wall thickness with preserved systolic function, but arrhythmias are present and are usually identified at diagnosis.

**LVNC with congenital heart disease**: pulmonic stenosis, pulmonary atresia, tricuspid atresia, and double outlet right ventricle are more frequently associated with LVNC than are other congenital heart lesions; septal defects and left-sided lesions are also common.

**Table S1**. A brief summary of the subtypes of LVNC

| **Subtypes** | **LV size** | **LV wall thickness** | **LVEF** |
| --- | --- | --- | --- |
| Benign LVNC | ─ | ─ | ─ |
| Dilated LVNC | ↑ | ─/↓ | ↓ |
| Hypertrophic LVNC | ─ | ↑, asymmetrical septal hypertrophy | ─/↑ |
| Hypertrophic dilated LVNC | ↑ | ↑, local hypertrophy | ↓ |
| Restrictive LVNC | left atrial or biatrial ↑, diastolic dysfunction | | |
| LVNC with arrhythmias | benign LVNC + arrhythmias | | |
| Right ventricular or biventricular LVNC | | | |
| LVNC with congenital heart disease | | | |

LVNC: left ventricular noncompaction; LVEF: left ventricular ejection fraction.

**Table S2.** Baseline Characteristics of All Subjects

| Characteristics | n (%)/mean ± SD | Characteristics | n (%)/mean ± SD |
| --- | --- | --- | --- |
| Age, y | 48.2±15.4 | Cardiovascular mortality | 14 (7) |
| BMI, kg/m^2^ | 23.16±3.02 | Echocardiography | |
| Time from onset to diagnosis, y | 2.52±3.89 | LVEF (%) | 48.17±16.34 |
| Male sex | 123 (61.5%) | LVEF≥50 | 92 (46) |
| Smoking | 57 (28.5%) | 40≤LVEF<50 | 29 (14.5) |
| Coronary heart disease | 34 (17) | LVEF<40 | 79 (39.5) |
| Stroke | 15 (7.5) | LVEDD, mm | 59.25±12.47 |
| Hypertension | 59 (29.5) | LVESD, mm | 44.60±14.71 |
| Diabetes mellitus | 25 (12.5) | LAD, mm | 36.00±8.42 |
| Dyslipidemia | 40 (20) | LV dilation | 129 (64.5) |
| Family history of CM/SCD | 8 (4) | LA dilation | 58 (29.2) |
| NYHA functional class III-IV | 55 (27.5) | Pulmonary hypertension | 42 (21) |
| Symptoms at presentation | | Mild | 24 (12) |
| Dyspnea | 132 (66) | Moderate | 14 (7) |
| Palpitation | 35 (17.5) | Severe | 4 (2) |
| Chest pain | 21 (10.5) | LV thrombosis | 9 (4.5) |
| Dizziness/Presyncope/syncope | 14 (7) | Cardiac magnetic resonance (n=133) | |
| Asymptomatic | 21 (10.5) | Compacta thickness, mm | 4.03±1.81 |
| Medical treatment | | Noncompacta thickness, mm | 14.35±7.50 |
| Beta blockers | 154 (77) | Maximum NC/C ratio | 3.19±1.06 |
| ACE inhibitor/ARB | 78 (39) | Segments of NC | 3.05±2.07 |
| ARNI | 85 (42.5) | Electrocardiogram |  |
| MRA | 112 (56) | Arrhythmias | 103 (51.5) |
| Diuretics | 108 (54) | LBBB | 21 (10.5) |
| Anticoagulation | 41 (20.5) | RBBB | 7 (3.5) |
| Aspirin | 50 (20) | AVB | 15 (7.5) |
| SGLT2 inhibitors | 13 (6.5) | Af/AF | 22 (11) |
| Cardiovascular implantable devices | | ventricular arrhythmias | 75 (37.5) |
| ICD | 4 (2) | VT/VF | 25 (12.5) |
| CRT | 10 (5) | ST-T abnormalities | 130 (65) |
| MACE | 47 (23.5) | LV hypertrophy | 29 (14.5) |
| HF hospitalization | 33 (16.5) | QTc, ms | 432.60±44.01 |
| All-cause mortality | 16 (8) | QTc prolongation | 59 (29.5) |

IQR indicates the 25th to 75th percentile interquartile range. BMI: body mass index; CM/SCD: cardiomyopathy/sudden cardiac death; NYHA: New York Heart Association; ACE: angiotensin-converting enzyme; ARB: angiotensin receptor blocker; ARNI: angiotensin receptor-neprilysin inhibitor: MRA: mineralocorticoid receptor antagonist; SGLT2: sodium-glucose cotransporter 2; MACE: major adverse cardiovascular events; HF: heart failure; LVEF: left ventricular ejection fraction; LVEDD: left ventricular end-diastolic diameter; LVESD: left ventricular end-systolic diameter; LAD: left atrial diameter; NC: noncompacted; C: compacted; LBBB: left bundle branch block; RBBB: right bundle branch block; AVB: atrioventricular block; QTc: corrected QT interval.

**Table S3.** Univariate Cox Analysis for the Variables Associated with MACE

| Variables | Hazard Ratio (95% CI) | *P* value |
| --- | --- | --- |
| Clinical variables | | |
| Age, y | 1.04 (1.02-1.06) | <0.001 |
| Male sex | 1.53 (0.80-2.93) | 0.194 |
| BMI, kg/m^2^ | 1.04 (0.95-1.14) | 0.378 |
| Time from onset to diagnosis, y | 1.06 (1.00-1.11) | 0.05 |
| Asymptomatic | 0.30 (0.04-2.17) | 0.232 |
| Smoking | 1.39 (0.76-2.53) | 0.284 |
| Coronary heart disease | 1.80 (0.94-3.42) | 0.076 |
| Stroke | 1.43 (0.56-3.63) | 0.458 |
| Hypertension | 1.42 (0.76-2.64) | 0.272 |
| Diabetes mellitus | 1.57 (0.76-3.25) | 0.227 |
| Dyslipidemia | 1.31 (0.65-2.63) | 0.458 |
| NYHA functional class III-IV | 1.74 (0.97-3.12) | 0.062 |
| Echocardiography | | |
| LVEF<50%  LVEF≥50%  40≤LVEF<50  LVEF<40% | 3.19 (1.53-6.64)  1.00  2.91 (1.15-7.35)  3.29 (1.54-7.03) | 0.002  0.024  0.002 |
| LVEF, % | 0.968 (0.95-0.99) | 0.002 |
| LVEDD, mm | 1.04 (1.01-1.07) | <0.001 |
| LVESD, mm | 1.04 (1.02-1.06) | <0.001 |
| LAD, mm | 1.07 (1.03-1.10) | <0.001 |
| LV dilation | 2.44 (1.03-5.80) | 0.043 |
| LA dilation | 2.32 (1.18-4.57) | 0.014 |
| Pulmonary hypertension | 3.24（1.81-5.82） | 0.006 |
| LV thrombosis | 3.68（1.44-9.40） | 0.007 |
| CMR (n=133) | | |
| Minimum compacta thickness, mm | 1.11 (0.82-0.50) | 0.512 |
| Maximum noncompacta thickness, mm | 1.03 (1.01-1.05) | 0.014 |
| Maximum NC:C ratio | 1.08 (0.62-1.70) | 0.836 |
| Segments of noncompaction | 1.33 (0.90-1.97) | 0.148 |
| Electrocardiogram | | |
| Arrhythmias | 1.55 (0.84-2.84) | 0.162 |
| Ventricular arrhythmias | 1.13（0.63-2.02） | 0.693 |
| Ventricular tachycardia/ fibrillation | 2.81（1.42-5.57） | 0.003 |
| LBBB | 2.06 (0.99-4.29) | 0.053 |
| Atrial flutter/fibrillation | 2.58 (1.34-4.95) | 0.004 |
| QTc prolongation | 1.77 (0.86-3.64) | 0.122 |

BMI: body mass index; NYHA: New York Heart Association; LVEF: left ventricular ejection fraction; LVEDD: left ventricular end-diastolic diameter; LVESD: left ventricular end-systolic diameter; LAD: left atrial diameter; NC: noncompacted; C: compacted; LBBB: left bundle branch block; QTc: corrected QT interval.

**Table S4**. Logistic Analysis for the Variables Associated with LV dysfunction

| Variable | Univariate Analysis | | Multivariable Analysis | |
| --- | --- | --- | --- | --- |
|  | Odds Ratio | *P* value | Odds Ratio | *P* value |
| Age, y | 1.04 (1.02-1.06) | <0.001 |  |  |
| Male sex | 0.81 (0.45-1.46) | 0.487 |  |  |
| BMI, kg/m^2^ | 1.06 (0.96-1.17) | 0.378 |  |  |
| Asymptomatic | 0.23 (0.07-0.75) | 0.014 |  |  |
| Smoking | 2.34 (1.20-4.54) | 0.012 |  |  |
| Coronary heart disease | 1.62 (0.75-3.52) | 0.22 |  |  |
| Hypertension | 1.09 (0.58-2.03) | 0.79 |  |  |
| Diabetes mellitus | 3.09 (1.18-8.13) | 0.022 |  |  |
| Dyslipidemia | 1.37 (0.68-2.78) | 0.385 |  |  |
| Mid-basal noncompaction | 3.18 (1.76-5.73) | <0.001 | 2.73 (1.28-5.86) | 0.01 |
| Arrhythmias | 2.47 (1.38-4.41) | 0.002 |  |  |
| LV hypertrophy by voltage criteria | 2.10 (0.84-5.26) | 0.114 |  |  |
| QTc prolongation | 8.67 (3.52-21.35) | <0.001 | 9.16 (3.55-23.66) | <0.001 |

BMI: body mass index; LV: left ventricular.
